# Efficacy and Safety of Combination Treatment With Apatinib and Osimertinib After Osimertinib Resistance in Epidermal Growth Factor Receptor-Mutant Non-small Cell Lung Carcinoma—A Retrospective Analysis of a Multicenter Clinical Study

**DOI:** 10.3389/fmolb.2021.639892

**Published:** 2021-05-05

**Authors:** Xue Yang, Yang Xia, Liyan Xu, Li Liang, Minglei Zhuo, Meina Wu, Tongtong An, Ziping Wang, Yuyan Wang, Jianjie Li, Jia Zhong, Hanxiao Chen, Bo Jia, Jingjing Wang, Jun Zhao

**Affiliations:** ^1^Key Laboratory of Carcinogenesis and Translational Research (Ministry of Education), Department of Thoracic Medical Oncology, Peking University Cancer Hospital and Institute, Beijing, China; ^2^Key Laboratory of Respiratory Disease of Zhejiang Province, Department of Respiratory and Critical Care Medicine, Second Affiliated Hospital of Zhejiang University School of Medicine, Hangzhou, Zhejiang, China; ^3^Department of Medical Oncology, Beijing Chest Hospital, Capital Medical University, Beijing, China; ^4^Department of Tumor Chemotherapy and Radiation Sickness, Peking University Third Hospital, Beijing, China

**Keywords:** osimertinib, apatinib, EGFR, NSCLC, resistance

## Abstract

Currently, there are limited treatment options for patients who developed resistance to osimertinib, a third-generation epidermal growth factor receptor (EGFR) inhibitor. Resistance to EGFR inhibitors is frequently associated with enhanced vascular endothelial growth factor (VEGF) levels. This multicenter, retrospective study aimed to evaluate the efficacy of the combination treatment with apatinib and osimertinib in 39 patients with EGFR-mutant non-small cell lung carcinoma (NSCLC) who developed osimertinib resistance. The patients received the combination of oral apatinib 250 mg qd and osimertinib 80 mg qd. The efficacy was evaluated after the first month then every 2 months thereafter. The primary endpoint was progression-free survival (PFS). The overall response rate (ORR) and the disease control rate (DCR) of the combination of apatinib and osimertinib was 12.8% (5/39) and 79.5% (31/39), respectively. The median PFS was 4 months [95% confidence interval (CI): 3.5–4.5 months]. Fourteen patients were administered with at least 6 months of combination therapy, and 11 of them remained on treatment programs. The 6-month PFS rate was 38%. Nine patients underwent biopsies after failing osimertinib treatment, and five of six patients with TP53 mutations had PFS of less than 3 months. The spectrum of resistance to osimertinib mechanisms included c-mesenchymal-epithelial transition factor (c-Met) amplification, phosphatidylinositol-4,5-bisphosphate 3-kinase catalytic subunit alpha (PIK3CA) gain-of-function mutation, phosphatase and tensin homolog (PTEN) loss-of-function mutation, Erb-B2 receptor tyrosine kinase 2 (ERBB2) amplification, and insulin-like growth factor 1 receptor (IGF1R) mutation. The most common adverse events were hypertension (30.7%, 12/39), diarrhea (15.4%, 6/39), and proteinuria (12.8%, 5/39). The combination of apatinib and osimertinib improved the ORR and the DCR of patients with osimertinib-refractory EGFR-positive NSCLC, thus making it a reasonable treatment choice after the development of osimertinib resistance.

## Introduction

Osimertinib, a third-generation epidermal growth factor receptor-tyrosine kinase inhibitor (EGFR-TKI), can selectively block mutations that are sensitive to EGFR-TKI. It is resistant to EGFR T790M mutation. In patients with non-small cell lung carcinoma (NSCLC) who had EGFR T790M mutations that are resistant to both first- and second-generation EGFR-TKIs, it can help achieve a median progression-free survival (PFS) of approximately 10 months (Mok et al., 2017). However, some patients are becoming resistant to osimertinib, which presents additional challenges. Currently, there are limited treatment options for patients who developed osimertinib resistance (Planchard et al., 2015; Ou et al., 2016; Zhou et al., 2019).

Resistance to EGFR inhibitors is frequently associated with enhanced vascular endothelial growth factor (VEGF) levels. Dual inhibition of the VEGF receptor (VEGFR) and EGFR signaling pathways shows the potential to overcome osimertinib resistance (Naumov et al., 2009; Larsen et al., 2011). Apatinib is an oral tyrosine kinase inhibitor that targets VEGFR-2 (Mi et al., 2010). We previously reported good efficacy and prolonged PFS benefit of using the combination of apatinib and a first-generation EGFR-TKI in cases of EGFR mutation-positive NSCLC (Li et al., 2017) progression. However, there are no data about the efficacy of the combination of a VEGFR inhibitor and osimertinib after third-generation EGFR-TKI failure. Thus, this study aimed to evaluate the efficacy of the combination of apatinib and osimertinib in patients with EGFR-mutant NSCLC who developed osimertinib resistance.

## Materials and Methods

### Study Population

**FIGURE 1:**
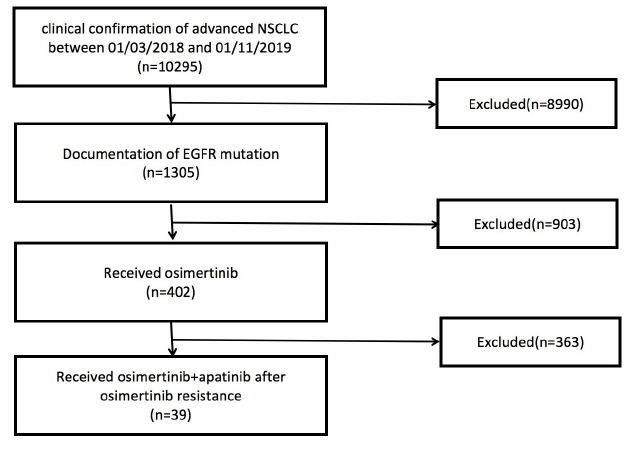
Study cohort selection. EGFR, epidermal growth factor receptor; NSCLC, non-small cell lung carcinoma.

In this analysis, 39 patients were enrolled from four participating institutions, namely, Peking University Cancer Hospital (*n* = 24), Second Affiliated Hospital of Zhejiang University School of Medicine (*n* = 9), Beijing Chest Hospital (*n* = 4), and Peking University Third Hospital (*n* = 2), between March 1, 2018 and November 1, 2019 (Figure 1). Every patient who displayed resistance to osimertinib during treatment for advanced lung adenocarcinoma was identified from the databases of the four institutions. All the patients received the combination of osimertinib and apatinib after progression of osimertinib resistance. None of the enrolled patients received other types of antiangiogenic therapy except for apatinib prior to or during combination therapy. This multicenter, retrospective study was approved by the ethics committee of the four participating institutions. All the patients provided informed consent for treatment in this protocol.

### Treatment Regimens

The patients received osimertinib at a dose of 80 mg and apatinib at a dose of 250 mg, taken orally once daily. The treatment would be discontinued in the event of disease progression, unacceptable toxicity, or if the physician or patient opted to end the treatment.

### Efficacy and Safety Assessments

Tumor response was radiographically evaluated according to RECIST guidelines version 1.1. Efficacy evaluation was conducted after the first month, then every two months thereafter. The primary endpoint was PFS, which is defined as the time from the first administration of the combination of osimertinib and apatinib to the first documentation of progression of disease according to RECIST guidelines version 1.1. The ORR was calculated as the sum of CR and PR rates, whereas the DCR was calculated as the sum of SD, CR, and PR rates. Toxicities during osimertinib and apatinib combination treatment were assessed according to the Common Terminology Criteria for Adverse Events (CTCAE) 4.0 classification.

### Capture-Based Targeted Next-Generation Sequencing

A subgroup of the patients (*n* = 9) enrolled in the study was subjected to several biopsies (liquid or tumor) once osimertinib resistance progressed and prior to the administration of apatinib. We obtained a biopsy of the tumor of a patient from a lesion, after which we performed targeted next-generation sequencing (NGS) according to previously described methods (Lin et al., 2018; Zhuo et al., 2020). Eight patients underwent liquid biopsy using targeted NGS as previously described (Zhuo et al., 2020). The collected samples of liquid biopsy included peripheral blood (*n* = 6), pleural effusion (*n* = 1), and cerebrospinal fluid (*n* = 1).

### Statistical Analysis

The Kaplan–Meier method was used to estimate the median PFS, while we produced 95% CIs with a log–log transformation. Safety analyses were done through descriptive methods as well as percentages. SPSS version 22.0 (IBM, Armonk, NY, United States) was used to calculate and produce the results of all statistical analyses performed in this study. *P* < 0.05 was considered statistically significant.

## Results

### Patient Characteristics

Of the 39 patients, 18 were male and 21 were female. Thirty patients were never smokers, while nine of them were current or former smokers. Every patient was a carrier of EGFR mutations; and according to their TKI-naïve samples, there were 16 exon 19 deletion, 17 exon 21 L858R mutation, and 6 exon 18 G719X mutation cases. In total, 4, 25, and 10 patients received osimertinib as first-, second-, and later-line treatment, respectively. The median PFS for osimertinib was 8 months (95% CI: 7.1–8.9). NGS data from 9 patients who developed osimertinib resistance were available for analysis. The clinical characteristics of the patients are listed in Table 1.

**TABLE 1:** Baseline clinical features of patients enrolled in the study (*n* = 39).

**Characteristic**	**Value**
**Sex**	
Male	18 (46%)
Female	21 (54%)
**Age (years)**	
Median	54
<65	31 (79%)
≥65	8 (21%)
**Smoking history**	
Yes	9 (24%)
No	30 (76%)
**ECOG performance status**	
≤1	31 (79%)
2	8 (21%)
**Stage**	
Iva	14 (36%)
IVb	25 (64%)
**Metastasis site**	
Brain	18 (46%)
Bone	17 (44%)
Liver	12 (31%)
**EGFR mutation status**	
EGFR exon 19 del	16 (41%)
EGFR exon 21 L858R	17 (44%)
EGFR exon 18 G719X	6 (15%)
**Osimertinib treatment**	
First line	4 (10%)
Second line	25 (64%)
Beyond second line	10 (26%)
**Previous treatment**	
Radiotherapy	5 (13%)
Chemotherapy	10 (26%)
**Osimertinib disease progression model**	
Intrathoracic progression	24 (62%)
Extrathoracic progression	15 (38%)

*ECOG, Eastern Cooperative Oncology Group; EGFR, epidermal growth factor receptor.*

### Molecular Characteristics of Post-osimertinib/Pre-combination Treatment Specimens

Rebiopsy for NGS was performed in nine patients during osimertinib treatment and prior to the combination treatment. We found that of all somatic variations in nine patients, mutations related to EGFR sensitivity were the most common. TP53 mutation was found in seven of nine patients (77.8%), whereas T790M mutation was identified in four of nine patients (44.4%). The spectrum of resistance to osimertinib mechanisms included c-Met amplification (*n* = 1), PIK3CA gain-of-function mutation (*n* = 1), PTEN loss-of-function mutation (*n* = 1), ERBB2 amplification (*n* = 1), and IGF1R mutation (*n* = 1).

### Outcomes of Combination of Apatinib and Osimertinib Treatment

**FIGURE 2:**
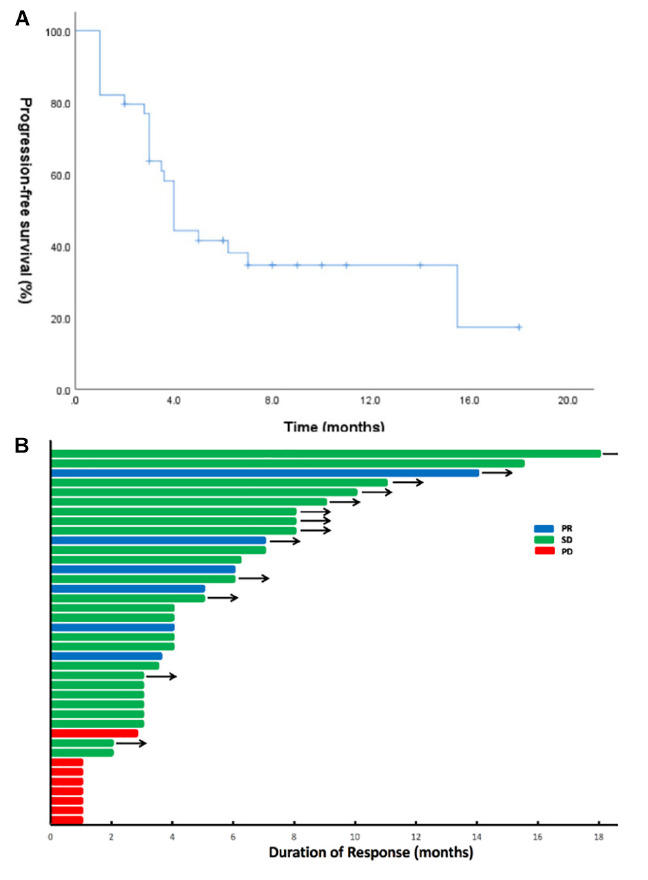
Apatinib and osimertinib activity in osimertinib-resistant EGFR-mutant NSCLC. **(A)** Progression-free survival (PFS) in the 39 patients treated with the combination of apatinib and osimertinib. **(B)** Swimmer plots showing the duration of combination treatment for each patient in the study cohort. Patients who developed a progressive disease are denoted in red, stable disease in green, and partial response in blue. Arrows indicate patients continuing on the combination of apatinib and osimertinib at the time of last follow-up.

**FIGURE 3:**
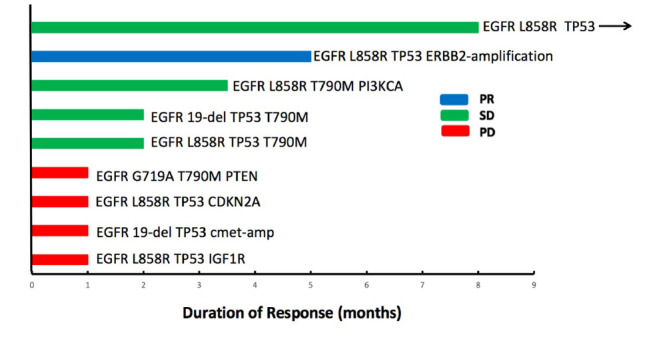
Individual duration of combined treatment in patients with post-osimertinib/pre-combined biopsies. Patients who developed a progressive disease are denoted in red, stable disease in green, and partial response in blue. Arrows indicate patients continuing on the combination of apatinib and osimertinib at the time of last follow-up.

**FIGURE 4:**
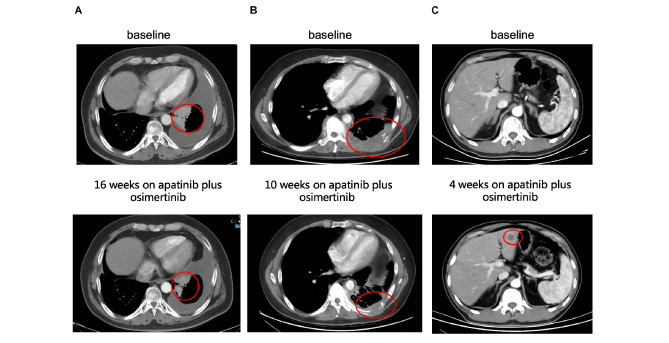
Examples of tumor response to the combination of apatinib and osimertinib in EGFR-positive cases with osimertinib resistance. **(A)** Partial response of a left lung mass to combination therapy in a patient with TP53 mutation detected post-osimertinib/pre-combined treatment biopsy. This patient remained on treatment at the time of last follow-up. **(B)** Partial response of a left chest wall mass to combination therapy in a patient with TP53 mutation and ERBB2-amplification detected post-osimertinib/pre-combined treatment biopsy. **(C)** Progressive disease demonstrated with new hepatic metastases after 1 month of combination treatment in a patient with TP53 and PTEN loss-of-function mutations detected in the post-osimertinib/pre-combined treatment biopsy.

Until the last follow-up on April 1, 2020, the disease progressed in 25 patients (64.1%), and the remaining 14 patients (35.9%) were still being treated with combination therapy (Figure 2). The median follow-up time was 9.8 months (range: 4.8–24.8 months). The ORR and the DCR for the combination of apatinib and osimertinib were 12.8% (5/39) and 79.5% (31/39), respectively. Median PFS was 4 months (95% CI: 3.5–4.5). Fourteen patients had received at least a 6-month combination therapy, and 11 of them were still on treatment. The 6-month PFS rate was 38%. The data are based on treatment length and patient response to the profiling of mutations after the development of osimertinib resistance and are shown in Figure 3. Among the 6 patients whose PFS less than 3 months, 5 had pathogenic mutations of TP53 and 3 had maintained T790M (Table 2). Examples of three cases with different resistance mechanisms demonstrated various therapeutic effects (Figure 4).

**TABLE 2:** Basic characteristics of nine patients with NGS testing.

**Patient No.**	**Gender**	**Age**	**Smoking history**	**ECOG**	**Stage**	**EGFR mutation**	**PFS of Osimertinib (months)**	**Osimertinib PD model**
1	M	61	No	1	IVa	L858R	8	Extrathoracic
2	M	62	No	2	IVb	19-del	8	Extrathoracic
3	F	55	No	1	IVa	L858R	12	Intrathoracic
4	F	44	No	1	IVb	G719A	6	Intrathoracic
5	F	54	No	1	IVa	L858R	2	Intrathoracic
6	F	66	No	2	IVa	19-del	11	Intrathoracic
7	M	35	No	1	IVb	L858R	6	Intrathoracic
8	M	62	No	1	IVb	L858R	12	Extrathoracic
9	F	53	No	1	IVb	L858R	8	Intrathoracic

The most frequently observed adverse events were hypertension (30.7%, 12/39), diarrhea (15.4%, 6/39), and proteinuria (12.8%, 5/39). Other less common adverse events included rash (*n* = 3), hand–foot syndrome (*n* = 3), hoarseness (*n* = 2), thrombocytopenia (*n* = 2), and liver dysfunction (*n* = 1). Notably, a patient achieved partial response; however, the combination therapy was terminated because of tachycardia and markedly decreased left ventricular ejection fraction (Table 3).

**TABLE 3:** Treatment-related adverse events in all patients (*n* = 39).

	Any grade*	Grades 1–2*	Grade 3*
Adverse event	35 (90%)	31 (79%)	4 (11%)
Hypertension	12 (31%)	10 (26%)	2 (5%)
Diarrhea	6 (15%)	6 (15%)	–
Proteinuria	5 (13%)	5 (13%)	–
Rash	3 (8%)	2 (5%)	1 (3%)
Hand–foot syndrome	3 (8%)	3 (8%)	–
Hoarseness	2 (5%)	2 (5%)	–
Thrombocytopenia	2 (5%)	2 (5%)	–
Liver dysfunction	1 (3%)	1 (3%)	–
Decreased left ventricular ejection fraction	1 (3%)	–	1 (3%)

*Grading per the Common Terminology Criteria for Adverse Events, version 4.0.

## Discussion

Significant research has been conducted on osimertinib resistance, and treatment strategies after osimertinib treatment failure have been evaluated (Le et al., 2018; Leonetti et al., 2019; Zhao et al., 2020). However, there are limited data on the use of apatinib after the development of osimertinib resistance. Given the broad range of resistance mechanisms currently emerging with osimertinib, the potential role of combination therapy in certain osimertinib-refractory settings warrants further investigation. This multicenter retrospective analysis demonstrated that treating patients with the combination of osimertinib and apatinib resulted in a 13% ORR and a median PFS of 4 months, together with a safety profile (Lin et al., 2018). In the scope of our knowledge, this is the first large-scale study to assess the clinical nature of administering combination therapy with osimertinib and apatinib under osimertinib-refractory conditions.

Resistance to osimertinib is related to genomic alterations that are both dependent and independent of EGFR and includes an acquired tertiary mutation related to EGFR resistance (EGFR C797S mutation), MET amplification, aberrations in downstream signaling pathways (mutations in the RAS and RAF genes), and epithelial–mesenchymal transition (Ricordel et al., 2018). For patients with EGFR C797S found alongside T790M, the PFS for the combination treatment of erlotinib and osimertinib was almost 3 months (Wang et al., 2017). Treatment using a combination of cetuximab and brigatinib showed promising results for patients with EGFR-T790M-*cis*-C797S mutations; however, in this study, only few patients were assessed.

To date, For MET-driven acquired resistance, the longest PFS for combination treatment with c-Met inhibitor and osimertinib has been almost 5 months (Sequist et al., 2020). Aside from these types of targeted therapy, the addition of antiangiogenic inhibitors to EGFR-TKI therapy after progression of EGFR-TKI therapy is also an attractive strategy. Preclinical and clinical evidence suggests that EGFR-TKIs may work synergistically with VEGF inhibitors (Naumov et al., 2009; Larsen et al., 2011). We previously showed that apatinib, together with gefitinib, displayed significant antitumor properties in patients with NSCLC because of EGFR-TKI resistance related to T790M, under both *in vitro* and *in vivo* conditions (Li et al., 2017). Moreover, a previous retrospective analysis of patients with advanced NSCLC who failed icotinib treatment showed that the combination of apatinib and icotinib reached a median PFS of 5.33 months (95% CI: 3.63–7.03 months) (Xu et al., 2017). Additionally, patients with EGFR-mutant NSCLC who were first treated with a combination therapy using apatinib and gefitinib demonstrated prolonged PFS inclinations, indicating that the therapy was a reasonable treatment (Zhang et al., 2019). Collectively, these findings support that treatments using the combination of apatinib and a first-generation EGFR-TKI is well tolerated and has good efficacy.

This study produced results similar to those of Liu et al., who assessed three patients afflicted with T790M-positive lung adenocarcinoma. After progression of osimertinib resistance, their patients responded to sustained therapy combining osimertinib and apatinib within a PFS range of 5–7 months (Liu et al., 2019). This suggests that the particular mechanism related to resistance could alter patient response to the apatinib and osimertinib treatment in osimertinib-refractory tumors. EGFR-mutant NSCLC could be affected by changes in TP53, which would result in reduced genomic stability. Previous studies have shown that concomitantTP53 mutations were correlated with lower survival rates in patients with EGFR alterations (Blakely et al., 2017). In our study, we also found that pathogenic TP53 mutation was the most common mutation after the development of osimertinib resistance. In addition to TP53 mutation, maintained T790M mutation was also associated with heterogeneous mechanisms of resistance (PI3KCA mutation and PTEN loss). This finding indicated that subclones with T790M mutants can be found alongside subclones with specific mechanisms related to resistance (Zhao et al., 2020). In this case, targeting T790M alone is unlikely to result in clinical benefit. Among the three of four patients who barely benefited from the combination therapy, with PFS of less than 1 month, T790M loss was related to the progression of other mechanisms related to resistance, such as c-Met amplification and IGF1R and CDKN2A mutations. Our findings underscore the value of conducting multiple biopsies (Lin et al., 2018) in patients who develop progression during osimertinib treatment. In addition, it is essential to explore novel biomarkers predictive of treatment response to the combination of osimertinib and apatinib in patients with osimertinib resistance.

This retrospective study had several limitations and lacked a control group. Moreover, while a multicenter analysis was performed to identify patient eligibility, selection bias was still a potential problem. Thus, further multicenter prospective research is required to validate the experiment results.

In summary, the combination of apatinib and osimertinib in osimertinib-refractory EGFR-positive NSCLC improved the ORR and the DCR. The results promote combination treatment therapy in patients with osimertinib resistance, especially in ones with a non-targetable resistance mechanism. Further studies are warranted to determine biomarkers predictive of treatment response to anti-VEGF therapy for patients with osimertinib-resistant EGFR-mutant NSCLC.

## Data Availability Statement

The data used to produce the results of this study are not publicly available due to ethical and privacy restrictions. However, the data are available from the corresponding author upon request.

## Ethics Statement

This multicenter, retrospective study was approved by the ethics committee of four participating institutions. All patients provided informed consent for treatment in this protocol.

## Author Contributions

XY and JZa: conception and design. XY, YX, LX, LL, MZ, MW, TA, ZW, YW, and JL: acquisition of data (provided acquired and managed patients, provided facilities, etc.). XY, JZo, HC, BJ, and JW: analysis and interpretation of data (e.g., statistical analysis, biostatistics). XY, YX, LX, LL, and JZa: writing, review, and/or revision of the manuscript. All authors contributed to the article and approved the submitted version.

## Conflict of Interest

The authors declare that the research was conducted in the absence of any commercial or financial relationships that could be construed as a potential conflict of interest. The reviewer JD declared a past co-authorship with several of the authors XY, JZo, HC, JZa, TA, and YW to the handling editor.

## Abbreviations

CR: complete response
CI: confidence interval
DCR: disease control rate
EGFR: epidermal growth factor receptor
EGFR-TKI: epidermal growth factor receptor-tyrosine kinase inhibitor
NGS: next-generation sequencing
NSCLC: non-small cell lung carcinoma
ORR: overall response rate
PR: partial response
PFS: progression-free survival
RECIST: response evaluation criteria in solid tumors
SD: stable disease
VEGF: vascular endothelial growth factor
VEGFR: vascular endothelial growth factor receptor.
